# Effect of Soil Moisture Regimes on Growth and Seed Production of Two Australian Biotypes of *Sisymbrium thellungii* O. E. Schulz

**DOI:** 10.3389/fpls.2018.01241

**Published:** 2018-08-28

**Authors:** Gulshan Mahajan, Barbara George-Jaeggli, Michael Walsh, Bhagirath S. Chauhan

**Affiliations:** ^1^Queensland Alliance for Agriculture and Food Innovation, The University of Queensland, Gatton, QLD, Australia; ^2^Department of Agronomy, Punjab Agricultural University, Ludhiana, India; ^3^Queensland Alliance for Agriculture and Food Innovation, The University of Queensland, Warwick, QLD, Australia; ^4^Agri-Science Queensland, Department of Agriculture and Fisheries, Warwick, QLD, Australia; ^5^Sydney Institute of Agriculture, School of Life and Environmental Sciences, The University of Sydney, Narrabri, NSW, Australia

**Keywords:** photosynthesis, ecology, proline, soluble sugar, water stress, drought adaptation, weed

## Abstract

*Sisymbrium thellungii* O. E. Schulz is an emerging problematic weed in the northern grain region of Australia. Several different biotypes exist in this region but not all biotypes exhibit the same growth and reproduction behavior. This might be due to local adaptation to the different agro-ecosystems, however, information on this aspect is limited. To determine whether adaptation to water stress was a factor in biotype demographic growth and reproduction behavior, we evaluated the physiological and biochemical responses of two Australian *S. thellungii* biotypes, selected from high (Dalby) and medium (St. George) rainfall areas, to different pot soil moisture levels corresponding to 100, 75, 50, and 25% of soil water holding capacity (WHC). Averaged across moisture levels, the St. George biotype (medium rainfall area) had 89% greater biomass and produced 321% more seeds than the Dalby biotype. The St. George biotype was less affected by increased levels of water stress than the Dalby biotype. The Dalby biotype produced 4,787 seeds plant^-1^ at 100% WHC and only 28 seeds plant^-1^ at 25% WHC. On the other hand, the St. George biotype produced 4,061 seeds plant^-1^ at 25% WHC and its seed production at 100% WHC was 9,834 seeds plant^-1^. On a per leaf area basis and averaged across all moisture levels, the St. George had significantly lower net carbon assimilation compared with the Dalby biotype, accompanied by a trend for lower stomatal conductance, which might indicate an adaptation to water stress. Across the moisture levels, the St. George biotype had higher phenolics and total soluble sugar, but free proline content was higher in the Dalby biotype compared with the St. George biotype. Like total soluble sugar, proline content increased with water stress in both biotypes, but it increased to a greater extent in the Dalby biotype, particularly at the 25% of WHC. Branching, flowering and maturity occurred earlier in the St. George biotype compared with the Dalby biotype, indicating relatively faster growth of the St. George biotype, which again seems to be an adaptation to water-limited environments. In conclusion, the St. George biotype of *S. thellungii* had higher reproductive capacity than the Dalby biotype across all the moisture regimes, which suggests greater invasiveness. Overall, the large size and rapid growth of the *S. thellungii* population from the medium rainfall area, together with its physiological response to water stress and its ability to maintain seed production in dry conditions, may enable this biotype to become widespread in Australia.

## Introduction

*Sisymbrium thellungii* O. E. Schulz is an emerging problematic C_3_ weed of the northern grain region of Australia, where it has evolved resistance to acetolactate synthase (ALS) inhibiting herbicides (Heap, 2018); therefore, herbicidal control of this weed winter crops like canola (*Brassica napus* L.), chickpea (*Cicer arietinum* L.) and wheat (*Triticum aestivum* L.) is challenging. For this reason, a better understanding of the biology of *S. thellungii* has become a priority for its proper management.

Weeds compete with crops for resources like water, light, and nutrients. Among them, water is the most limiting factor for attaining optimum yield in any crop (Brown, 1995). Changes in cultural practices and rapid adaptability to climate change frequently favor the dominance of weeds in agro-ecosystems (Mahajan et al., 2012). Modeling studies on climate change have shown that rising temperatures in Australia would lead to an increase in the frequency of droughts and a reduction in rainfall events (DEE, 2017). It has been observed that crops in the northern grain region of Australia sometimes experience severe drought, resulting in weed abundance and yield loss (Parreira et al., 2015). Limited soil water availability influences crop-weed competition to a significant degree, and in general, weed growth is favored due to the greater plasticity of weeds as compared to crops (Crusciol et al., 2001; Chauhan and Mahajan, 2014; Mahajan et al., 2015). Drought plays an important role in weed invasion by affecting weed physiology and weed ecology (Bajwa et al., 2016). The availability of soil moisture determines whether weeds will establish and subsequently spread (Chauhan and Johnson, 2010). Weed species and even weed biotypes collected from different environmental conditions can vary in their response to soil moisture.

Higher atmospheric CO_2_ levels as a result of global warming, may reduce stomatal conductance and transpiration in plants, thereby lowering latent heat loss and causing higher leaf temperature (Bernacchi et al., 2007). Besides stomatal control of photosynthesis, maintenance of a low level of photosynthesis and its early recovery related to conductance and water potential suggests that the non-stomatal control of photosynthesis is an important attribute of some drought-tolerant weeds (Hill and Germino, 2005).

Thus, in the future, plants including weeds are likely to experience increases in acute heat and drought stress, and if weeds are more robust to these conditions this can be expected to negatively influence crop productivity and promote weed invasiveness (Thomas et al., 2004). However, compared to crops, the literature related to the response of weeds to water stress is limited. Weeds or weed biotypes able to produce a high number of seeds at low soil moisture content exhibit drought tolerance and may pose tough competition to crops in a water-scarce environment. Such drought tolerance in weeds could be due to the maintenance of turgor by means of osmoregulation, increased elasticity of cells, decreased cell size, or desiccation tolerance through protoplasmic resistance (Sullivan and Ross, 1979). Osmoregulation in plants maintains turgor, which reduces the effect of water stress on physiological functions such as stomatal opening, photosynthesis and growth (Chimenti et al., 2002). Many weeds survive by having low osmotic potential before stress, prolonging the maintenance of turgor and delaying the leaf rolling mechanism (White et al., 1992; Mundree et al., 2002). Proline content in several desert weeds [e.g., *Haloxylon ammadendron* C. A. Mey., *Zygophyllum xanthoxylum* (Bunge) Engl., and *Artemisia sphenocephala* Krasch.] suggests that free proline accumulation may underpin their adaptation to arid environments. Many authors have found that the strong antioxidant system of weeds helps in alleviating the impact of radical oxygen species, generated by metabolic processes in response to water stress (Lu et al., 2008).

The effect of drought on plant phenology is variable and depends upon the plant species, as well as the timing, duration and extent of drought. Phenological responses to water stress are considered important drought avoidance mechanisms in plants (Stout and Simpson, 1978; Muchow and Carberry, 1989). Information is very limited on variations of phenological stages in weeds in response to water stress. Even under limited soil moisture conditions, it has been observed that some weeds and weed biotypes exhibited strong capacity to complete their life cycle, maintain growth and produce sizable amounts of seeds (Chauhan and Johnson, 2010; Kaur et al., 2016). Studies on summer annual weeds, such as *Amaranthus rudis* Sauer (Sarangi et al., 2016), *Rottboellia cochinchinensis* (Lour.) (Chauhan, 2013) and *Amaranthus palmeri* S. Wats. (Moran and Showler, 2005) showed that water stress negatively affected growth; however, the effects of soil moisture levels on growth and reproduction of winter annual weeds have not been tested. Therefore, an experiment was conducted to compare the effect of different soil moisture regimes on *S. thellungii* growth, physiological and biochemical changes, and its reproductive capacity. Such parameters could be helpful in evaluating and differentiating the invasive capacity of *S. thellungii* under future water-scarce climate scenarios.

## Materials and Methods

### Experimental Design

**FIGURE 1:**
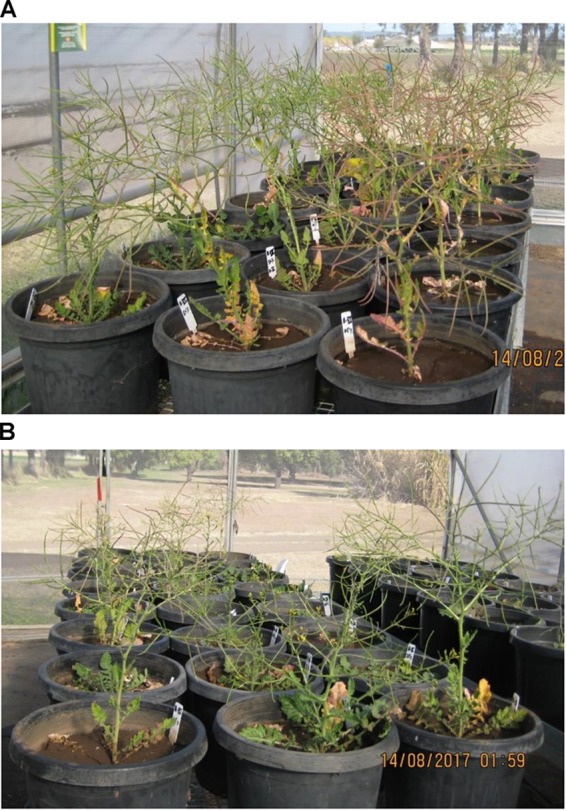
St. George biotype **(A)** and Dalby biotype **(B)** of African turnip under different water regimes in naturally ventilated greenhouse.

**FIGURE 2:**
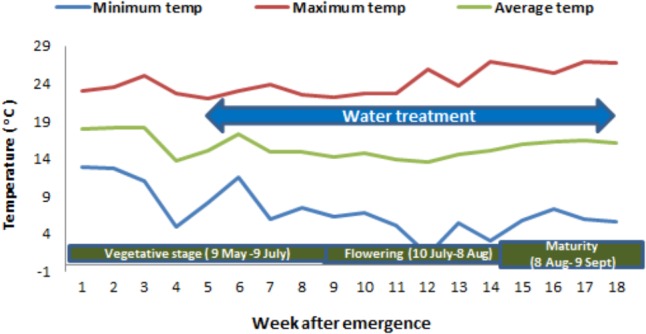
Weekly mean maximum, minimum and average temperature (°C) during different stages of African turnip weed.

The experiment was conducted in a completely randomized design with eight treatments (2 biotypes × 4 soil moisture levels) in a factorial arrangement. Plants were grown in large pots in a naturally ventilated greenhouse at the Gatton Campus of the University of Queensland, Australia, during the winter season of 2017 (**Figure 1**). The weekly average temperature during the vegetative stage, flowering and maturity period of the plants varied from 13.8 to 18.1°C, 13.6 to 14.8°C, and 14.6 to 16.4°C, respectively (**Figure 2**). The optimum temperature for germination of African turnip weed is 20/10°C (day/night) but no information is available on the optimum temperature range for its growth. This experiment was conducted in the winter season, in which this weed grows in the field. The temperature in the naturally ventilated greenhouse was similar to the ambient temperature.

Two Australian biotypes of *S. thellungii* (St. George and Dalby) were grown at four different soil moisture levels, as determined by soil gravimetric water holding capacity (WHC): 100, 75, 50, and 25%. Each treatment had six replications. The St. George biotype was collected from a medium rainfall area with an average annual rainfall of around 515 mm. The Dalby biotype was sourced from a high rainfall area with an average annual rainfall of 680 mm^1^. Three seeds were sown per pot and after emergence, seedlings were thinned to a single plant per pot. The soil moisture levels were maintained throughout the study period as per treatments. The soil, collected from the Gatton Research Farm, was a heavy clay loam with a pH of 6.7, an electrical conductivity of 0.14 dSm^-1^ and an organic matter content of 2.8%. The modified method of Chauhan and Johnson (2010) and Nguyen (2011) was used for calculating WHC. Three pots (30 cm diameter) containing approximately 13.5 kg of soil were saturated with tap water. The pot surface was then covered with a plastic container and the pots were allowed to drain for 48 h. Thereafter, from the middle of each pot, three soil samples (each *ca.* 300 g) were taken. These samples were weighed (wet weight of soil, A), oven-dried (90°C for 72 h) and re-weighed (dry weight of soil, B). The WHC was then calculated using the formula: [(A – B) × 100]/B. The 75, 50%, and 25% WHC were estimated based on that fraction of the WHC. The water treatments were started 40 days after sowing (DAS) as the initial growth of the weeds was slow. A measured quantity of water as per treatment was applied every other week after the start of the treatments. The 1-week interval for water application at 100% WHC (field capacity) was standardized based on soil moisture meter reading.

For the initial 1 month of establishment, water was applied every alternative day to keep the pots moist. The experiment ran for 124 days until the plants were fully matured and seed production had ceased.

### Growth, Phenology, Photosynthetic Parameters, and Seed Production

At peak vegetative growth (75 DAS), plant height in each pot was measured from the soil surface to the uppermost tip of the plant and all leaves on each plant were counted. The number of days after sowing to the appearance of the first branch and initiation of the first flower was recorded for each plant. Photosynthetic parameters were measured on healthy, fully expanded and undamaged leaves at 99 DAS to coincide with the biochemical analysis. Plants of all six replicates were measured; however, several plants could not be measured as their leaves were too small which resulted in incomplete replicates. Gas exchange measurements were taken between 1000 and 1300 h using a LI-6400 portable photosynthesis system (LI-COR Inc., Lincoln, NE, United States) with a 6400-40 leaf chamber fluorometer which measures 2 cm^2^ of leaf. Measurements were taken on a random rosette leaves at steady state controlling the following parameters: 300 μ mol^-1^ s^-1^ air flow to the sample, 1500 μmol quanta m^-2^ s^-1^ irradiance (PAR), 400 μmol mol^-1^ reference CO_2_ and 27°C leaf temperature. The vapour pressure deficit (VPD) based on leaf temperature at the time of measurement ranged from 0.9 to 2.0 kPa.

At harvest, aboveground-biomass and root biomass (70°C for 72 h) were determined separately. Total seed number was estimated by multiplying the silique number with the average seed number of five randomly chosen siliques per plant.

### Biochemical Analysis

Undamaged, healthy, fresh, and penultimate leaves (*ca.* 3 g) were taken from three replicates of each treatment at 100 days after sowing. The samples were stored at 4°C in zip lock polyethene bags until used for analysis *ca*. 7 days later. From these samples, the soluble phenolics were determined by following the Folin–Ciocalteu reagent method (Julkenen-Titto, 1985). The total soluble sugar content of each sample was determined by following the procedure of Dubios et al. (1956) modified according to Lee and Kim (2000). Free leaf proline content in each sample was measured by following the procedure of Bates et al. (1973).

### Statistical Analyses

Growth, phenology and biochemical data were analyzed using analysis of variance (ANOVA) to evaluate differences between treatments (GENSTAT 16th edition; VSN International, Hemel Hempstead, United Kingdom). Means were separated using Fisher’s protected least significant difference (l.s.d.) test at *P* = 0.05.

Due to missing data causing an unbalanced design, the photosynthetic parameter data were analyzed using the lme4 package in R (Bates et al., 2015) fitting a linear mixed model of the form *y* = Xβ + Z μ + e with Biotype and Water Regime fitted as fixed (vector β) and Replicate as random (vector μ) effects and e denoting an error term. The initial model included interactions between biotype and water regime, but this term was dropped as it was not significant for either variable presented here.

Figures were prepared using SigmaPlot software (SigmaPlot 13; Systat Software, San Jose, CA, United States) and R (R Core Team, 2016).

## Results

### Growth Parameters and Phenology

By 75 DAS, the St. George and Dalby biotypes attained similar plant height at 100% WHC, however, plant height of the Dalby biotype was reduced with decreasing soil moisture levels (*P* < 0.05). Plant height of the St. George biotype was only reduced at the most severe treatment of 25% WHC (**Table 1**). By 75 DAS both biotypes had similar numbers of leaves at 100% WHC and leaf number was reduced at 25% WHC for both populations (**Table 1**). Water stress delayed branching and flowering initiation in both biotypes, but flowering was particularly affected in the Dalby biotype (*P* < 0.05) (**Table 1**). In the St. George biotype, plants started branching and flowering 34 and 28 days later, respectively, at 25% WHC compared with plants grown at 100% WHC. At this low WHC, branching and flowering of the Dalby biotype was delayed by 33 and 53 days, respectively, compared to plants at 100% WHC.

**Table 1:** Effect of different soil moisture levels (factor 2) on final plant height, leaf number at 75 days after sowing and days taken to branching and flowering initiation of two Australian biotypes (St. George and Dalby; factor 1) of *Sisymbrium thellungii*.

Moisture level	St. George	Dalby	*Mean*
**Plant height (cm)**			
100% WHC	56.7a	51.7a	54.2a^∗^
75% WHC	53.7a	34.0d	43.8b
50% WHC	65.4b	31.4d	48.4b
25% WHC	44.2c	24.2e	34.2c
Mean	55.0a	35.3b	
LSD (*P* ≤ 0.05)	Biotype = 4.8; Water regime = 6.8; B × WR = 9.6		
**Leaf number (75 DAS)**			
100% WHC	27a	28a	28a
75% WHC	26a	24a	25a
50% WHC	28a	20a	24b
25% WHC	20a	16a	28a
Mean	25a	22b	
LSD (*P* ≤ 0.05)	Biotype = 2.6; Water regime = 3.7; B × WR = NS		
**Days taken to branching**			
100% WHC	63a	68bc	65.5a
75% WHC	66ab	71cd	68.5ab
50% WHC	70bcd	73d	71.5b
25% WHC	97e	96e	96.5c
Mean	74a	77b	
LSD (*P* ≤ 0.05)	Biotype = 2.3; Water regime = 3.2; B × WR = 4.5		
**Days to flower initiation**			
100% WHC	68a	67a	67.5a
75% WHC	71a	94b	82.5b
50% WHC	73a	105b	89.0b
25% WHC	96b	120c	108c
Mean	77a	96.5b	
LSD (*P* ≤ 0.05)	Biotype = 6.6; Water regime = 9.4; B × WR = 13.3		


WHC, Water holding capacity; LSD, Least significant difference; B, Biotype; NS, Not significant; WR, Water regime. Within terms Biotype (B), Water Regime (WR), B × WR, means followed by identical letters are not significantly different at α = 0.05. ^∗^ indicates means value were compared with LSD of respective factor and thus, letters for each factor are separate.

### Biomass and Seed Production

Averaged across moisture levels, the St. George biotype (medium rainfall area) had 89% greater biomass and produced 321% more seeds than the Dalby biotype (**Table 2**). Increasing soil moisture stress affected biomass and seed production negatively in both biotypes. Averaged over biotypes, 25% WHC reduced *S. thellungii* biomass by 41% and seed production by 72% compared to 100% WHC. While there was no significant interaction between biotype and water regime for either trait, biomass of the St. George biotype, remained similar at 100, 75, and 50% WHC. Seed production of this biotype was also less affected by water stress than the Dalby biotype. At 100% WHC, the St. George biotype produced 9,834 seeds plant^-1^, while the Dalby biotype produced approximately half of that (4,787 seeds plant^-1^) (**Table 2**). However, at 25% WHC, the seed production of the Dalby biotype was reduced to only 28 seeds plant^-1^ while the St. George biotype produced 4,061 seeds plant^-1^. Water regime had no significant effect on the root to shoot biomass ratio; however, the root to shoot ratio was generally higher in the Dalby biotype compared with the St. George biotype (**Table 2**).

**Table 2:** Effect of different soil moisture levels (factor 2) on different parameters of two Australian biotypes (St. George and Dalby; factor 1) of *Sisymbrium thellungii*. Moisture level

	St. George	Dalby	Mean
**Biomass (g plant^-1^)**			
100% WHC	9.64a	5.59a	7.61a
75% WHC	9.21a	4.45a	6.83a
50% WHC	9.01a	4.14a	6.58a
25% WHC	5.30a	3.62a	4.46b
Mean	8.29a	4.45b	
LSD (*P* ≤ 0.05)	Biotype = 1.22; Water regime = 1.72; B × WR = NS		
**Seeds plant^-1^ (number)**			
100% WHC	9834a	4787a	7310a
75% WHC	9175a	1781a	5478b
50% WHC	7797a	730a	4264b
25% WHC	4061a	28a	2044c
Mean	7717a	1831b	
LSD (*P* ≤ 0.05)	Biotype = 1357; Water regime = 1919; B × WR = NS		
**Root: Shoot ratio (dry weight)**			
100% WHC	0.08a	0.15a	0.11a
75% WHC	0.13a	0.12a	0.12a
50% WHC	0.12a	0.23a	0.17a
25% WHC	0.15a	0.19a	0.17a
Mean	0.12a	0.17b	
LSD (*P* ≤ 0.05)	Biotype = 0.05; Water regimes = NS; B × WR = NS		


WHC, Water holding capacity; LSD, Least significant difference; B, Biotype; NS, Not significant; WR, Water regimes. Within terms Biotype (B), Water Regime (WR), B × WR, means followed by identical letters are not significantly different at α = 0.05.

### Biochemical Attributes

Averaged across all moisture levels, the Dalby biotype had more than double the amount of free proline in leaves compared with the St. George biotype (**Table 3**). The amount steadily increased with increasing water stress and was almost six times higher at 25% compared with 100% WHC, while increasing water stress had no consistent effect on free proline levels in the St. George biotype. On the other hand, water-soluble carbohydrate content was significantly higher in the St. George biotype than the Dalby biotype and it gradually increased in both biotypes with increasing water stress, thus in both biotypes, water-soluble carbohydrate was significantly higher at 25% than at 100% WHC. The amount of soluble phenolics was 27% higher in the St. George biotype relative to the Dalby biotype; however, moisture level had no effect on soluble phenolics content in either biotype (**Table 3**).

**Table 3:** Effect of different soil moisture levels (factor 2) on different parameters of two Australian Biotypes (St. George and Dalby; factor 1) of *Sisymbrium thellungii*. Moisture level

	St. George	Dalby	Mean
**Free proline (μ moles g^-1^ dry weight)**			
100% WHC	0.61a	1.26a	0.91a
75% WHC	2.29a	2.33a	2.31a
50% WHC	1.86a	2.42a	2.14a
25% WHC	0.38a	7.58b	3.90a
Mean	1.29c	3.39d^∗^	
LSD (*P* ≤ 0.05)	Biotype = 1.58; Water regime = NS; B × WR = 3.17		
**Water soluble carbohydrate % (dry weight)**			
100% WHC	17.4a	14.0a	15.7a
75% WHC	18.2a	12.9a	15.6a
50% WHC	20.5a	13.7a	17.1a
25% WHC	20.8a	17.7a	19.3b
Mean	19.2a	14.6b	
LSD (*P* ≤ 0.05)	Biotype = 1.84; Water regime = 2.60; B × WR = NS		
**Total phenolics (mg gallic acid equivalent g^-1^ dry weight)**			
100% WHC	19.6a	14.1a	16.8a
75% WHC	14.7a	13.4a	14.1a
50% WHC	17.6a	14.0a	15.8a
25% WHC	16.7a	12.6a	14.6a
Mean	17.1a	13.5b	
LSD (*P* ≤ 0.05)	Biotype = 1.92; Water regime = NS; B × WR = NS		


WHC, Water holding capacity; LSD, Least significant difference; B, Biotype; NS, Not significant; WR, Water regime. Within terms Biotype (B), Water Regime (WR), B × WR, means followed by identical letters are not significantly different at α = 0.05. ^∗^ indicates means value were compared with LSD of respective factor and thus, letters for each factor are separate.

### Photosynthetic Parameters

**FIGURE 3:**
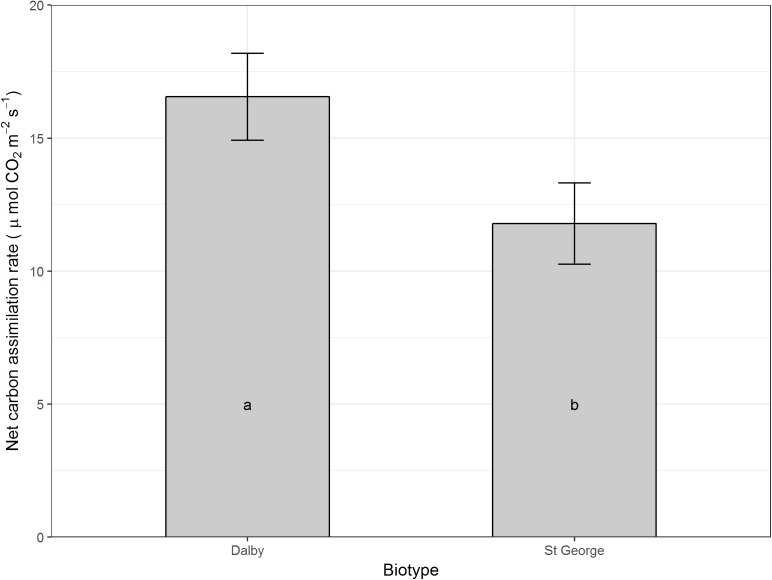
Net carbon assimilation rate (μmol CO_2_ m^-2^ s^-1^) of rosette leaves of two Australian biotypes (St. George and Dalby) of *Sisymbrium thellungii.* There were no significant interactions between biotype and water regime so data shown are means for each biotype across all treatments predicted from the linear mixed model. Vertical lines represent standard error and letters within columns indicate whether differences were significant at α = 0.5.

**FIGURE 4:**
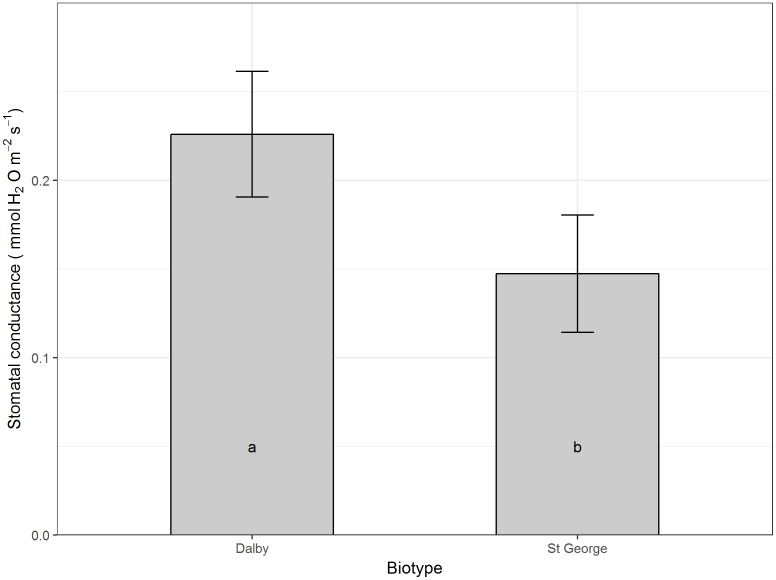
Stomatal conductance (mol H_2_O m^-2^ s^-1^) of rosette leaves of two Australian biotypes (St. George and Dalby) of *Sisymbrium thellungii.* There were no significant interactions between biotype and water regime so data shown are means for each biotype across all treatments predicted from the linear mixed model. Vertical lines represent standard error and letters within columns indicate whether differences were significant at α = 0.5.

Moisture level did not seem to affect net carbon assimilation or conductance on a per leaf area basis, however, averaged across all moisture levels and compared with the Dalby biotype, the St. George biotype had a reduced leaf net carbon assimilation (*P* > 0.05, **Figure 3**), which was accompanied by a trend for lower stomatal conductance (**Figure 4**, *P* = 0.066).

## Discussion

In the current study, we exposed two biotypes of *S. thellungii* O. E. Schulz collected from a high (Dalby) and a medium rainfall area (St. George) to different levels of water stress. Significant differences were observed between these two biotypes when grown under varied soil moisture stress. The St. George biotype exhibited more vigorous growth than the Dalby biotype and had higher reproductive output (seed yield) than the Dalby biotype under all levels of water stress. Most importantly, this population still produced high seed yield (4061 seeds plant^-1^) even under the highest level of water stress while the Dalby biotype yielded less than 30 seeds plant^-1^.

The increased growth of the St. George biotype was not reflected in increased net carbon assimilation on a per leaf area basis of rosette leaves. Rather, the St. George biotype had low rosette leaf carbon assimilation rates which were accompanied by a trend for lower stomatal conductance compared with the Dalby biotype, which might be a specific adaptation to low soil moisture. Down regulation stomatal conductance and carbon assimilation rates in older large rosette leaves while maintaining persistent whole-plant carbon assimilation and growth via photosynthetic stems and cauline leaves has been found in other weeds that are particularly well adapted to hot and dry environments (Hill and Germino, 2005).

Free proline is known to accumulate in plants as a result of water stress (Bates et al., 1973). The damaging effects of reactive oxygen species might be ameliorated by proline under moisture stress, helping plants to regulate physiological function (Bajwa and Farooq, 2016). Free proline levels were significantly and progressively increased in the Dalby biotype in response to decreasing soil moisture levels, but not in the biotype from St. George. In comparison to the Dalby biotype, the free proline content in the St. George biotype did not increase with increasing moisture stress. At 25% WHC, the proline content in the Dalby biotype was significantly higher than at 100% WHC.

The higher soluble sugar and soluble phenolics content in the St. George biotype in comparison to the Dalby biotype may have helped this biotype alleviate the effects of water stress, resulting in more vigorous growth and greater seed production. Elevated levels of these chemicals have been observed in various weed species in response to multiple abiotic stresses and they have been found to ameliorate the damaging effects of reactive oxygen species produced under moisture stress (Anjum et al., 2011; Bajwa et al., 2016, 2018). Also, soluble phenolics produced by some weeds have been reported to have allelopathic potential which further increases their competitiveness (Kanchan and Jayachandra, 1980; Bajwa et al., 2016).

The present study revealed that *S. thellungii* biotypes selected from high and medium rainfall areas have different growth and reproductive behavior. In the wake of climate change, it is projected that the frequency and severity of droughts in Australia will increase (DEE, 2017). This may have a negative effect on agriculture as water limitation during crop growth may provide weeds a competitive advantage (Patterson, 1995). Weed species or biotypes that have a fast growth habit, high biomass production and high reproductive potential will be more competitive than slow-growing species (Horak and Loughin, 2000); but again the competition might be dependent on level of water stress and needs further investigation.

## Conclusion

Our results demonstrated that the two Australian *S. thellungii* biotypes differed in growth, physiology, reproduction and biochemical production (free proline, total phenolics, and water-soluble carbohydrates) under well-watered as well as water stress conditions. The St. George biotype of *S. thellungii* had higher reproductive capacity than the Dalby biotype across all the moisture regimes, which suggests greater invasiveness. Overall, the large size and rapid growth of the *S. thellungii* population from the medium rainfall area, together with its physiological response to water stress and its ability to maintain seed production in dry conditions, may enable this biotype to become widespread in Australia.

While it has been known that certain biotypes show differences in competitiveness in different crop production systems, the physiological basis of these differences have not been shown previously. A better understanding of the underlying mechanisms of specific adaptation for each weed species is a prerequisite to develop the best management solutions for each agro-ecological region. With increasing frequencies of droughts, management solutions for biotypes with greater adaptation to drought are particularly important. With climate change, the St. George biotype of *S. thellungii* may expand its invasion range, and knowledge of its response to various soil moisture levels will become important in combating this weed.

Our study forms an excellent basis for such attempts for *S. thellungii*, an emerging and economically important weed in Australia. Information on the biological attributes of both biotypes under water stress conditions may be used to evaluate the effects of water limitation on weed-crop interactions in different areas via crop-modeling.

## Author Contributions

GM and BC designed the study, ran the experiment and wrote the paper. BG-J took the gas exchange measurements and wrote the physiological part. MW provided help in writing the manuscript. All authors read and approved the paper.

## Conflict of Interest Statement

The authors declare that the research was conducted in the absence of any commercial or financial relationships that could be construed as a potential conflict of interest. The reviewer DM and handling Editor declared their shared affiliation.

## Footnotes

1 http://www.bom.gov.au
